# Polyamines and Gut Microbiota

**DOI:** 10.3389/fnut.2019.00016

**Published:** 2019-02-25

**Authors:** Rosanna Tofalo, Simone Cocchi, Giovanna Suzzi

**Affiliations:** ^1^Faculty of Bioscience and Technology for Food, Agriculture and Environment, University of Teramo, Teramo, Italy; ^2^Farmacie Comunali di Romano di Lombardia, Bergamo, Italy

**Keywords:** polyamines, gut microbiome, human health, probiotics, fermented foods

The microbiota of gut is the community of microbes living in an individual's gastrointestinal tract. Several bacterial genera and species act in a concerted manner to establish metabolic interactions with the host (1). Although there is a general high interest in the study of metabolite flow across the microbe-host, at present, only some studies are targeting specific metabolites produced by intestinal microbiota such as polyamines (PAs) (2).

## Polyamines and Gut Microbiota

Polyamines can be defined as small polycationic molecules with a wide array of biological functions including gene regulation, stress resistance, cell proliferation and differentiation, and are associated to both eukaryotic and prokaryotic cells (3).

In human cells spermine, spermidine, and putrescine are the main PAs. Putrescine is produced in the cytoplasm of cells by decarboxylation of ornithine catalyzed by the enzyme ornithine decarboxylase (ODC). Spermine and spermidine are synthesized by S-adenosyl-methionine decarboxylase (AdoMetDC) and a transferase enzyme, catalyzing the transfer of the aminopropyl group to the primary amine group of putrescine or spermidine, respectively (4). The ingested food is the major source of PAs in the lumen, and the upper parts of intestine adsorb the majority of these compounds for growth processes throughout the body (5). The gut microbiota is considered the main responsible of PAs level in the lower part of intestine (6). Polyamines in the colonic lumen are transferred into the bloodstream via the colonic mucosa (7). Intracellular PAs levels are regulated by endogenous biosynthesis, degradation and exogenous transport. Both endocytic and solute carrier-dependent mechanisms have been described for polyamine uptake in the gut lumen (8). In eukaryotic cells they are involved in several physiological functions since they are able to bind to several anionic macromolecules such as DNA, RNA, proteins, and acidic phospholipids (9). The PAs involvement in maintaining chromatin structure and membrane stability and regulating ion-channels and scavenging free radicals has also been reported (10), as well as their role as second messengers in protein and nucleic acid synthesis for normal cell division and growth (11). In particular, cellular PAs availability contributes to tissue homeostasis of the gastrointestinal mucosa, the rates of epithelial cell division and apoptosis, by modulating the expression of various growth-related genes (12). In general, PAs are involved in several important cellular processes and their disregulation can affect growth, aging and several diseases such as cancer, neurodegeneration and metabolic disorders (13). To maintain good intracellular PAs contents, biosynthetic and catabolic processes are activated and highly regulated. For example, a high intracellular PAs levels are related with cell growth, whereas the inhibition of ODC decreases cellular PAs (12). On the contrary, its overexpression induces an increased level of PAs in human gut, a result that has been related with gastrointestinal cancers (14).

As regards bacteria, new putative phyla (134) other than the traditional ones (30) have been identified using culture-independent metagenomic sequencing and single-cell sequencing (15). However, the studies of PAs distribution in bacteria have been limited to culturable species and few bacterial species have been studied (16).

The bacterial PAs include spermidine, homospermidine, norspermidine, putrescine, cadaverine, and 1,3-diaminopropane with putrescine and spermidine being the most common PA (17, 18). Furthermore, because of high bacterial diversity, some microorganisms produce sym-homospemidine rather than spermidine or produce only a diamine and some bacteria do not produce PAs, such as *Staphylococcus aureus* (19).

Different bacterial species, up to 1,000, constitute the intestinal microbiota. This community of microorganisms (bacteria, archaea, fungi, protozoa, viruses) is responsible for the metabolism of non- digested food components and it can supply to the host nutrients such as amino acids and vitamins and other biologically active substances (20). In general, this microbial consortium is subject to fluctuations due to different factors such as environment, diet, disease states and many others (21). The microorganisms colonizing the gut can contribute to the overall health of the host or be pathogenic, invading the host, and causing diseases under certain conditions (22).

The majority of the studies on gut microbiota are focused on bacteria, even if all the biota plays important roles in host health and disease (23). The high-throughput sequencing techniques based on the amplification of the 16S rRNA identified more than 120 different prokaryotic *phyla* with only 31 *phyla* included cultured species (24). Moreover, the majority of species that constitute the gut microbiota belong mainly to four *phyla*: *Firmicutes, Bacteroidetes, Actinobacteria, Proteobacteria*, and among these *Firmicutes* and *Bacteroidetes* are dominant *phyla* in agreement with the high-throughput sequences carried out the last 10 years. The dominant species belong to the families *Bacillaceae, Enterobacteriaceae, Corynebacteriaceae*, and *Bacteroidaceae*, with a prevalence of anaerobic species, uncultured yet (25). Culturomics is a new strategy that could improve the study of microorganisms of the human gut microbiota (26).

As regards *Archaea*, non-methanogenic and methanogenic species are present in human gut microbiota, with the latter producing methane during anaerobic fermentations. These species belong to *Euryarchaeota* phylum. A very small fungal community is present in the human gut with three major phyla: *Ascomycota* 63%, *Basidiomycota* 32%, and *Zygomycota* 3%.

**Figure 1:**
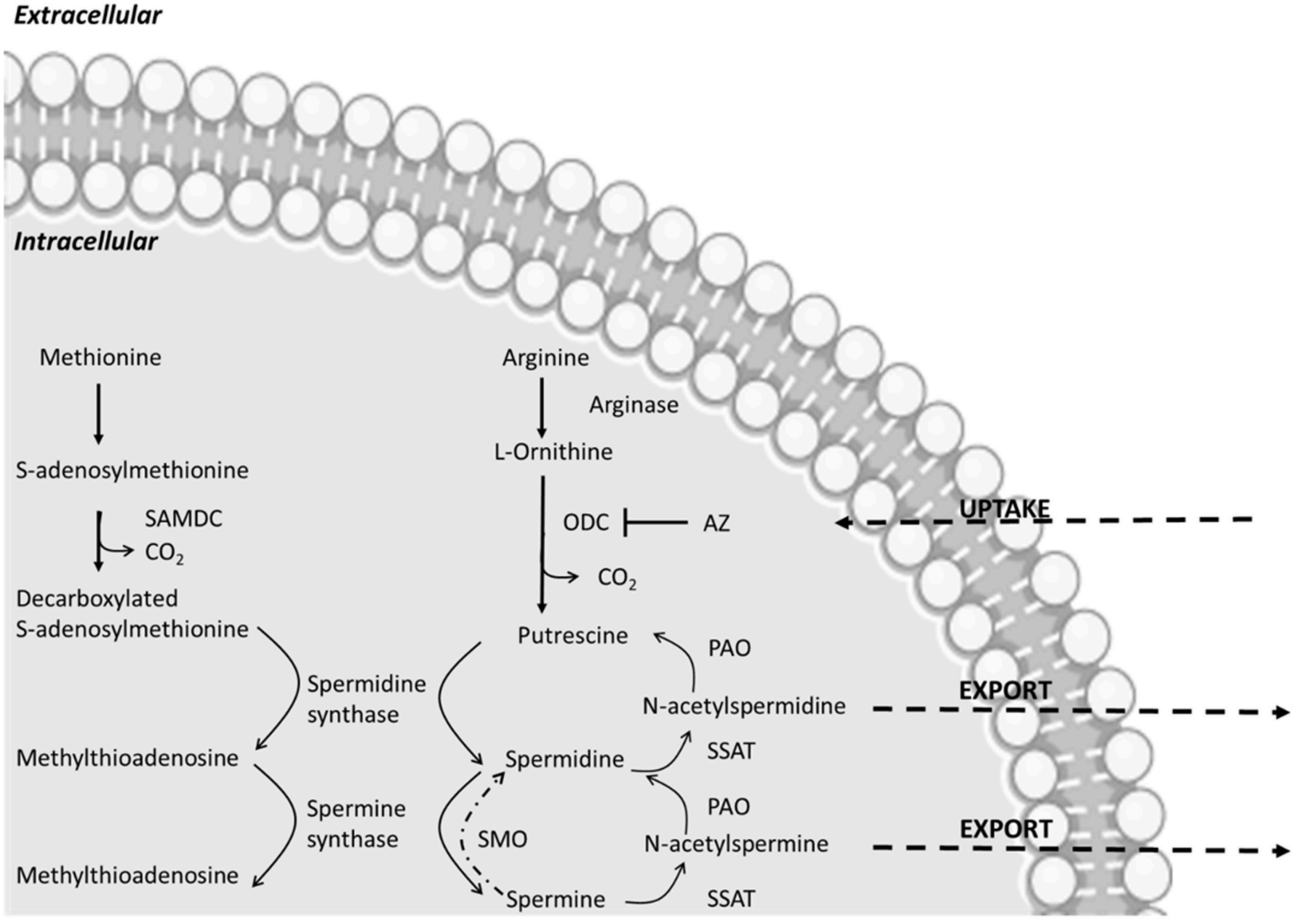
Main polyamine metabolic pathways modified from Linsalata et al. (27). The abbreviations used are as follows: ODC, ornithine decarboxylase; SAMDC, S-adenosylmethionine decarboxylase; SSAT, spermidine-spermine-N-acetyl transferase; PAO, polyamine oxidase; AZ, antizyme; SMO, spermine oxidase. AZ inactivates ODC, increases polyamine efflux and decreases polyamine uptake.

Putrescine, cadaverine, spermidine, and spermine are the main PAs encountered in bacteria (Figure 1). Their synthesis is highly regulated at molecular level through a concerted biosynthesis and uptake mechanisms, as well as by degradation and efflux processes. Their production relies on the presence of amino acidic precursor or other intermediates which are then converted into functional PAs (28). Besides *de novo* synthesis pathways, PAs uptake can be controlled through specific transport systems. They are highly conserved among bacteria. The best-known examples are two ABC transporters described in *Escherichia coli* that are specific for either putrescine or spermidine and two antiporters, exchanging putrescine for ornithine and lysine for cadaverine (29).

Recent studies highlighted the involvement of PAs in bacterial pathogenesis. A clear example is *Shigella* spp. an intracellular pathogen associated to enteric syndrome in humans (30). For instance, due to mutations and deletions, cadaverine is lost from *Shigella* spp., improving the pathogenicity process, because cadaverine has a protective effect on intestinal mucosa from enterotoxins. Spermidine accumulation increases *Shigella* resistance to oxidative stress and its survival in macrophages [for a review see (28)].

## Contribution of Gut Microbiota to PAs Formation

Almost all foods contain PAs; they are abundant in soybeans, mushrooms, wheat germ, beef, pork, chicken livers, oranges, turban shell viscera, and green tea leaves. A great part of PAs introduced by foods is absorbed in the small intestine, whereas microbiota produce these compounds in great amounts in the large bowel (2). Little is known about the production and degradation of biogenic amines (BAs) by gut microbiota and in particular PAs. Recently, isolates from the human gut, belonging to many different species, were found to produce and degrade BAs at different levels depending on the strains (16). Putrescine and spermidine, important metabolites of intestinal bacteria, are present in the intestinal lumen in concentrations ranging from 0.5 to 1 mM in healthy humans (6). Gut microorganisms can synthesize putrescine, spermine and spermidine, that are present at millimolar concentrations, and play a major role in providing PAs for the high demand of these compounds in intestine. Bacteria use PAs for cell to cell communication, cellular signals and cell differentiation and the bacterial metabolism of these compounds determines the PAs intestinal content. The main studies were performed in *E. coli*, even if it is a minor microbial component in the human intestine and its PAs biosynthetic pathway seems to be different from those present in dominant gut microbiota (31). Few studies report data on metabolites produced by intestinal microbiota and their functions, in particular short fatty acids (32) and PAs (33, 34). In addition, *Bacteroides* spp. and *Fusobacterium* spp. can synthesize putrescine and spermidine *in vitro* and *in vivo* (35). Recently Nakamura et al. (20) found that in colonic lumen putrescine is produced by different bacteria from collective biosynthetic pathways depending on a complex exchange of metabolites.

The environmental *stimuli* can modulate the gut microbiota metabolism as well as the absorption and release of PAs. Noack et al. (36) reported that indigestible polysaccharides pass into the large intestine and are fermented with the production of short-chain fatty acids and lower pH, that can modify the intestinal microbiological metabolism and composition, and stimulate intestinal PAs content synthesis. In general, the fermentable carbohydrates present in the large bowel contribute to increase the bacterial PAs formation with consequent beneficial effects on the gut mucosa. In addition, by using *in silico* analysis, novel PAs biosynthetic and transport proteins have been found. There are few studies on PAs biosynthetic pathway carried out by dominant intestinal microorganisms. In fact, great part of gut bacteria utilizes carboxyspermidine dehydrogenase and carboxyspermidine decarboxylase (CASDC) for spermidine biosynthesis, whereas *E. coli* utilizes S-adenosylmethionine decarboxilase and spermidine synthase (8). The species in the genus *Bacteroides*, which is predominant in intestine of humans with 20 of 56 most abundant species, harbors CASDC homologs (31) that are essential for spermidine biosynthesis contributing to the normal bacterial growth (37). Sugiyama et al. (8) evaluated the capacity of 32 bacterial species dominant in human gut to produce PAs in the cell and in supernatants, suggesting the presence of new genes and transporters. As many colonic microbial species do not possess complete synthetic pathways to produce PAs (38), it is possible to suppose the existence of metabolic interactions among bacterial species in the gut. Kitada et al. (39) showed that putrescine concentration produced by a mixed culture of different microbial species from gut microbiota was higher than that obtained with the single cultures. They demonstrated that a mixed culture of *E. coli* and *Enterococcus faecalis* produced the highest quantities of putrescine when the pH of the medium drops to neutral, suggesting the involvement of bacterial acid resistance system (40). A new pathway for putrescine formation was identified, from arginine to agmatine, with the cooperation of these two species. In presence of low pH, the acid resistance system of *E. coli* produces agmatine from arginine, and an arginine—agmatine antiporter exchanges extracellular arginine for the intracellular end product of decarboxylation, agmatine (41). *Enterococcus faecalis*, through an agmatine/putrescine antiporter, metabolizes the agmatine to putrescine by agmatine deiminase pathway, yielding ATP, CO_2_, and NH_3_ (42). The presence of other bacteria, such as *Bifidobacterium* spp. producing acid compounds in gut, favors the induction of this new pathway for putrescine production (39, 43, 44). In fact, many intestinal species do not possess a complete synthetic pathway for putrescine production from arginine (45) and therefore, it is possible to suppose the existence of a metabolic pathway spanning multiple bacterial species in the gut (8, 39). However, the knowledge about the contribution of gut microbiota to PAs formation is scarce and not sufficient.

## The Next-Generation Probiotic Bacteria and Polyamines

There is an enormous amount of research on probiotics and their beneficial impact on human health. Probiotics are defined by Boirivant and Strober (46) as “live microorganisms that, when administrated in adequate amounts, confer a health benefit on the host.” The main sources of probiotics are gut or some fermented foods, such as kefir grains and yogurts, with *Lactobacillus* spp. and *Bifidobacterium* spp. being the most used microorganisms. *Saccharomyces boulardii, Bacillus* spp., *E. coli*, enterococci, and *Weissella* spp. are also included. With the development of new methodologies, a new era in probiotic research is started and the new probiotics are referred to next generation probiotics. In fact, there is an increasing interest in the use of gut commensal bacteria as potential probiotics, such as the genera *Bacteroides, Clostridium, Bifidobacterium*, and *Faecalibacterium* that predominate in the human gut microbiome (47). The mechanisms of probiotics activity are not clearly understood, even if many studies have been carried out (48). The potential biological effects of probiotics are characterized by an extremely diverse range, such as the new functional activities that are currently studied (49).

The species of colonic PAs-producing bacteria are several and different. The PAs concentration in the gut depends on the high or low presence of PAs-producing bacteria and also of PAs-absorbing bacteria. However, the presence of probiotics can increase the concentration of PAs in intestinal lumen as reported after the consumption of yogurt added with probiotics such as *Bifidobacterium animalis* subsp. *lactis* LKM512 (34). The consumption of yogurt containing the probiotic strain *B. animalis* subsp. *lactis* LKM512, increases the PAs concentration in human gut, favoring several positive effects for improving intestinal health, increasing lifespan and quality of life (33, 50, 51). PAs have been associated with cancer risk and represent a specific marker for neoplastic proliferation. The administration of probiotic *Lactobacillus rhamnosus* strain GG has been found to affect the synthesis of PAs in gut and the proliferation rates of gastric cell cancer. A relationship between PAs biosynthesis and probiotic action in carcinogenesis and cancer growth was found (52).

The consumption of probiotic strain *B. animalis* subsp. *lactis* LKM512, colonizes the colon and alters the intestinal microbiota, producing PAs. This alteration in intestinal microbiota favors some bacteria and suppress others, such as *Enterobacteriaceae* species, and *Enterococcus* spp. The produced PAs induce maintenance and/or recovery of intestinal barrier function and other beneficial activities such as longevity (53). The activation of PAs biosynthesis is performed by indigenous gut microbiota stimulated by environmental acidification induced by *Bifidobacterium*. In fact, these microorganisms do not possess enzymes involved in PAs biosynthesis (39).

A study carried out with a cocktail of probiotics, administered for 60 days, enhanced the PAs biosynthesis in canine inflamed colonic mucosa, regulating PAs levels (54). The administration of mixed probiotic cultures of *Lactobacillus* spp. strains has been described to induce positive health effects (55). Therefore, the positive effects have been proved in live and dead probiotic preparations [for a review see Adams (56)].

## Conclusion

Microbiota-generated metabolites are an essential part of human physiology and are generated through microorganism–microorganism and host–microorganism interactions, with profound effects on human health and disease. Among the metabolites generated by bacteria in human gut PAs exhibit various beneficial effects, such as increased longevity, recovery of injured mucosa, and favorable effects on cognitive function. However, there is limited knowledge of how microorganisms interact with each other to synthesize metabolites in gut such as PAs. To obtain these tools it will be important to analyse the individual species and strains within these communities including uncultured microorganisms. Future researches on next-generation probiotics and/or mixed cultures of probiotic species should be investigated in order to better understand human health problems in the intestinal tract and find new strategies to face them. PAs modulation by gut microbiota and probiotic consortia could be a good strategy to achieve beneficial effects for human health.

## Author Contributions

All authors listed have made a substantial, direct and intellectual contribution to the work, and approved it for publication.

### Conflict of Interest Statement

The authors declare that the research was conducted in the absence of any commercial or financial relationships that could be construed as a potential conflict of interest.
